# Is Myelodysplasia a Consequence of Normal Aging?

**DOI:** 10.1007/s11912-021-01136-5

**Published:** 2021-11-04

**Authors:** Sonja Heibl, Reinhard Stauder, Michael Pfeilstöcker

**Affiliations:** 1 Department of Internal Medicine IV, Klinikum Wels-Grieskirchen, Wels, Austria; 2 Paracelsus Medical University, Salzburg, Austria; 3 Department of Internal Medicine V, Hematology and Oncology, Comprehensive Cancer Center Innsbruck (CCCI), Medical University of Innsbruck, Innsbruck, Austria; 4 3rd Medical Department, Hanusch Hospital, H.Collinstr 30, 1140 Vienna, Austria

**Keywords:** Myelodysplastic syndromes, Aging, CHIP, Elderly, Clonality, Myeloid neoplasia

## Abstract

**Purpose of Review:**

To review available data on the relationship of MDS and aging and to address the question if biological changes of (premature) aging are a prerequisite for the development of MDS.

**Recent Findings:**

Whereas the association of MDS with advanced age and some common biologic features of aging and MDS are well established, additional evidence for both, especially on the role of stem cells, the stem cell niche, and inflammation, has been recently described.

**Summary:**

Biologically, many but not all drivers of aging also play a role in the development and propagation of MDS and vice versa. As a consequence, aging contributes to the development of MDS which can be seen as an interplay of clonal disease and normal and premature aging. The impact of aging may be different in specific MDS subtypes and risk groups.

## Introduction

Myelodysplastic syndromes (MDS) are hematopoietic stem cell disorders characterized by ineffective hematopoiesis resulting in peripheral blood cytopenias. MDS typically occur at an advanced age with a median age at diagnosis of 68 to 75 years [1–3]. The total incidence of about 4/100,000 per year increases dramatically with advanced age to approximately 40/100,000 per year at the age of ≥ 70 years [4] and to 50/100,000 per year at the age of ≥ 80 years [3]. MDS as clonal disorders may be preceded by a state of clonal hematopoiesis (CHIP) in which MDS defining features cannot (yet) be substantiated [5, 6].

Whereas CHIP-specific somatic mutations are rarely detected in persons younger than 40 years, they reveal an increasing incidence with advanced age as described in the landmark study published by Jaiswal et al. [7]. The presence of clonal hematopoiesis increases the risk both of hematologic malignancies and of vascular diseases (coronary heart disease and ischemic stroke) which at least partially explains an increased all-cause mortality in this subgroup of patients [7]. Besides clonal hematopoiesis associated with advanced age, other biologic features of aging may influence the initiation and propagation of age-associated diseases such as MDS. This review describes recent developments in deciphering the biology of aging and of MDS with a focus on potential interactions and synergies to finally address the question if biological changes of normal aging are a prerequisite for the development of MDS.

## Biology of Aging

### Aging

It is defined as a multi-dimensional process characterized by decreased reserves in physical function, in cognition, and in physiological integrity. Impaired resistance to stressors such as acute or chronic illnesses, infections, or psychosocial distress results in increased vulnerability [8, 9].

These individuals are at high risk of falls, disability, hospitalization, and adverse outcomes upon treatment. Clinically important manifestations of the aging process are the so-called geriatric syndromes which include frailty, falls, dementia, osteoporosis, depression, distress, fatigue, urinary incontinence, failure to thrive, or sarcopenia. Especially in cancer patients, frailty influences diagnostic procedures as well as treatment [10].

The definition and operationalization of frailty follow two concepts: according to the Fried criteria, frailty is defined as a clinical syndrome, the so-called frailty phenotype, which is defined by the presence of the symptoms of weakness, self-reported exhaustion, weight loss, and low physical activity [8]. In contrast, the model developed by the group of Rockwood et al. defines frailty as an accumulation of deficits. Based on this concept, the clinical frailty scale (CFS) has been developed to assess the degree of frailty [11–13].

A progressive loss of physical functions ahead of time is called premature aging, which is often associated with inflammation, oxidative stress, and immune dysfunction. Mitochondrial dysfunction seems to promote premature aging associated with a reduced life span [14, 15]. Short telomere syndromes are accelerated aging syndromes affecting primarily organs with increased cell turnover such as bone marrow, skin, lungs, and gastrointestinal tract. *DKC1*, *TERT*, and *TERC* are the most commonly affected genes leading to bone marrow failure and idiopathic pulmonary fibrosis as frequent manifestations [16].

Some of the fundamental biochemical and molecular processes in the aging have been characterized and depicted as the so-called hallmarks of aging [17]. Several of these biological mechanisms by itself contribute to the phenotype of older persons and to age-associated diseases. Remarkably, these processes are similar to some of the mechanisms essential in cancerogenesis [18].

### Genomic Instability

During the lifespan of individuals, exogenous and endogenous damage causes the accumulation of genetic damage. The DNA damage theory of aging argues that naturally occurring DNA damages plays a causal role in aging. The relevance of genomic instability is highlighted by the association of impairment in DNA repair and progeroid syndromes such as Werner syndrome or Bloom syndrome. Analyses of progeroid syndromes are fundamental in the understanding of underlying mechanisms as they represent rare genetic disorders which imitate physiologic aging resulting in a phenotype of premature aging [19].

### Cellular Senescence and Telomere Attrition

Genomic integrity is also maintained by mechanisms which keep the length and the function of telomeres. The length of telomeres determines the proliferative capacity of cells and forms the basis for the so-called replicative senescence. The relevance of telomere attrition is highlighted by its relevance in normal and premature aging and the delay of aging in experimental models by the stimulation of telomerase activity [20].

### Epigenetic Alterations

Current evidence supports the assumption that epigenetic changes namely of methylation of DNA or acetylation and methylation of histones play a role in aging [21]. Epigenetic alterations may result in progeroid syndromes as well. A relevant aspect of epigenetic alterations is that they may be principally reversible by drugs such as histone deacetylase inhibitors or histone acetyltransferase inhibitors [22].

### Alternative RNA Splicing

It allows the generation of a variety of variant protein isoforms. Alterations in splicing have been observed in healthy aging and in a variety of progeroid syndromes. Associated with senescence and aging, splicing defects occur in accelerated aging. Changes in the activity of splicing factors and in the production of key splice variants can impact cellular senescence and the aging phenotype [23].

### Mitochondrial Pathways

These impact a variety of cellular functions such as the production of reactive oxygen species (ROS), cellular signaling, and regulation of apoptosis. Disruption of mitochondrial function is a common denominator of healthy aging and of many age-related diseases. Namely, the redox balance has a profound impact on aging as highlighted by the free radical theory [24•].

### Stem Cell Exhaustion

It is a further feature of older age. Although hematopoietic stem cell numbers may increase with advanced age, stem cell renewal capacity and their ability to differentiate deteriorates and increased numbers cannot compensate for functional impairments [25•]. Accordingly, aging-induced damage of stem cell functions may play a key role in the pathophysiology of the various aging-associated disorders [26]. Causes and mechanisms of hematopoietic stem cells (HSC) aging include many age- and MDS-associated mechanisms such as DNA damage, increase of ROS, and epigenetic changes [27]. In hematologic research, recent concepts describe a heterogeneous pool of HSCs that differ in their capacity for self-renewal and differentiation. While lymphoid HSCs are diminished as a reflection of a declining immune system, myeloid HSCs increase in number throughout aging—observed as myeloid skewing—with diminished function [28, 29]. Thus, during lifetime-specific HSC pools, these are clonally selected [30]. Targeted deletions of important genome maintenance regulators in animal models were used to support the hypothesis of stem cell exhaustion leading to age-related phenotypes [31]. For stem cells, the functioning interaction of HSCs and the stem cell niche, i.e., the bone marrow microenvironment, is essential.

### Stromal Niche

Stem cells exert their action in the context of the microenvironment that per se may be altered in aging. Current studies reveal a connection between the bone marrow microenvironment and aging. Age-associated changes of the bone marrow microenvironment include alterations in the cellular composition, e.g., a notable increase of adipocytes, and a decline of mesenchymal stem cell proliferative capacity [32]. Alteration of the bone marrow microenvironment is leading to the dislocation of HSC from endosteal niches to non-endosteal niches, vascular remodeling, and changes in inflammation. The crosstalk between HSCs and the microenvironment is an important mechanism for the aging process of the hematopoietic system [33•]. Cytokines and their receptors may facilitate stroma—HSC interactions: in support of this concept, IL-6 and TGFß1 have been proposed to be involved in microenvironmental sensing, thus acting as regulators of hematopoietic aging [34]. In mouse transplantation models, defects of stem cell homing and an increase in mobilization associated with aging were observed [27, 35•].

In addition, the importance of the niche has been shown in recent experiments demonstrating the restrainment of rejuvenated HSC by an aged microenvironment [36]. Thus, extrinsic and intrinsic changes trigger the transition from young to aging HSC and possibly also from normal to malignant stem cells [32].

### Cellular Senescence and Inflammaging

The number of senescent cells increases with aging, which may reflect the diminished capacity to eliminate these potential oncogenic cells. These cells reveal alterations in the secretion of pro-inflammatory cytokines referred to as “senescence-associated secretory phenotype” [20]. These changes in immune cell signaling are part of another aging-associated feature, namely altered intercellular communication. Whereas this mechanism may be beneficial in the compensation of cellular damage, this pro-inflammatory secretome may contribute to aging. This state of subclinical smoldering systemic inflammation represents a relevant alteration in altered cellular communication and has thus been termed inflammaging [37]. It may result from tissue damage causing cellular senescence, pro-inflammation, enhanced increased activation of the NF-kB transcription factor, and occurrence of an impaired autophagy response. Thus, enhanced activation of the NLRP3 inflammasome and other pro-inflammatory pathways finally leads to increased production of IL-1b, tumor necrosis factor, and interferons [38]. This type of inflammation is often termed sterile inflammation as it reveals a shift from infectious to non-communicable causes [37, 38]. Inflammaging has been suspected to form the basis for many common age-associated disorders such as metabolic syndrome, type 2 diabetes, cardiovascular disease, cancer, depression, autoimmune diseases, neurodegenerative diseases, sarcopenia, and osteoporosis [38]. An evaluation of 147 patients older than 70 years with a newly diagnosed hematologic malignancy demonstrated an association of malnutrition, inflammation, and pronounced impairments including fatigue, depression, comorbidities, and reduced functional capacities. Clustering of these parameters may suggest a common underlying pathway [39].

### Deregulated Nutrient Sensing

The relevance of nutrient sensing is supported by the observation of pronounced increases in lifespan upon dietary restriction. It may be modulated by lifestyle modification such as the Mediterranean diet and by pharmacological interventions. Nutrient-sensing pathways (mTOR, IGF-1) play an essential role in aging processes. Lifestyle modifiable factors may be identified and may be promising for future trials [17, 40].

### Impaired Proteostasis

The failure to refold or degrade unfolded proteins has been linked both to aging and to age-related neurodegenerative diseases such as Alzheimer’s disease or Parkinson’s disease. Impaired proteostasis thus represents a common feature of advanced age termed the aging proteostasis decline [41].

### Cancer and Aging

They clearly share several common mechanisms, namely genomic instability, telomere attrition, epigenetic alterations, mitochondrial dysfunction, cellular senescence, stem cell exhaustion, and altered intercellular communication. The now well-defined role of age-dependent changes in inflammation is now reflected as an “enabling feature” to the hallmarks of cancer in addition to genomic instability [18]. Two further hallmarks of aging—loss of proteostasis and deregulated nutrient sensing—may be more relevant in the aging processes although “deregulated metabolism” has been included as an emerging hallmark of cancer. In contrast, the malignant proliferation of cancer seems to be a unique mechanism that once initiated works also independently from age.

Many of the fundamental biologic processes of aging may also be observed in the development of the myeloid neoplasia MDS. Some similarities have been known for long times, whereas others such as the observations on innate immunity in MDS have only been recently unveiled [42]. Specific features may be more prominent in MDS or specific MDS subtypes such as the relevance of mitochondrial dysfunction and ROS [43].

## Development of MDS

MDS are clonal myeloid disorders arising from neoplastic hematopoietic stem cells [5]. Several pre-MDS conditions have been described in recent years—for definitions of these conditions, see Table 1. Idiopathic cytopenia of unknown significance (ICUS) is characterized by a persistent cytopenia in one or more cell lineages not yet meeting the diagnostic criteria for MDS [5, 6].

**Table 1:** Definitions of pre-MDS conditions and MDS (adapted from 5): *ICUS*, idiopathic cytopenia of unknown significance; *CHIP*, clonal hematopoiesis of indeterminate potential; *CCUS*, clonal cytopenia of unknown significance

ICUS	Peripheral cytopenia, MDS criteria not fulfilled, no MDS-related mutations, no or only mild cytopenia (< 10%)
CHIP	No peripheral cytopenia, MDS criteria not fulfilled, no MDS-related mutations, no or only mild dysplasia (< 10%), blasts < 5%
CCUS	Peripheral cytopenia, MDS criteria not fulfilled, one or more MDS-related mutations, no or only mild dysplasia (< 10%), blasts < 5%
MDS	Peripheral cytopenia with no evidence of any other underlying cause, BM failure, substantial dysplasia in one or more cell lineages (at least 10% dysplastic cells in any lineage), blasts 5–19%, (not mandatory: MDS-associated chromosomal abnormalities or MDS-associated somatic mutations)

Clonal hematopoiesis of indeterminate potential (CHIP) is defined by the presence of a somatic mutation in a leukemia-associated driver gene with a variant allele frequency (VAF) ≥ 2% but the absence of peripheral blood cytopenia [44]. The combination of a WHO-defined cytopenia and a mutation in an MDS-associated gene is referred to as clonal cytopenia of unknown significance (CCUS) [45].

When establishing a diagnosis of CHIP or CCUS, overt MDS needs to be excluded according to diagnostic criteria, though the distinction between CCUS and MDS may be difficult in daily practice. The presence of dysplastic features or MDS-related criteria changes the diagnosis of CCUS to MDS. A subset of patients with these premalignant conditions progresses to MDS or AML. Individuals with CHIP exhibit a tenfold increased risk of developing a malignant hematologic disease with a yearly rate of 0.5 to 1% per year [46]. Patients with CCUS have the highest risk of progression to MDS being 80 to 90% at 5 years, depending on the type of mutation (*U2AF1*, *ZRSR2*, *SRSF2*, *JAK2*, *RUNX1*) as well as on the number of mutations. In patients with single mutations of *TET2*, *DNMT3A*, or *ASXL1*, the risk of progression to MDS seems to be lower at about 50% at 5 years [45]. Alterations in genes involving cell cycle regulation such as p53 may have detrimental effects in MDS patients and have been shown to be associated with other age-related diseases and parental life span [45].

## Pathophysiology of MDS—How Much Aging?

In MDS, a multitude of different mechanisms have been described that affect hematopoiesis: in individual patients, distinct combinations of pathophysiologic changes lead to dysregulated hematopoietic differentiation, which causes morphological dysplasia and peripheral cytopenias. Whereas MDS originate in the hematopoietic stem cell, the disease is a complex interplay of alterations of hematopoietic stem cells, the bone marrow microenvironment, and the immune system [42]. Meanwhile, cytogenetic aberrations and based on new technologies such as NGS, molecular changes are well defined in MDS. Both affect prominent regulatory processes of hematopoiesis [47–49]. Biological processes altered by these genetic changes include DNA methylation, chromatin modification, RNA splicing, cohesion formation, regulation of transcription, signaling, and DNA repair [45].

### Genomic Instability

It is the main driver event for MDS initiation and propagation by the accumulation of somatic mutations in hematopoietic stem cells and their progenitors [42]. This feature is not exclusive to MDS but has been described in the context of aging as well [19]. Genes involved in DNA repair and genomic stability are linked to and discussed to be causal for progeroid syndromes; therefore, somatic mutations could be important drivers of aging. Besides MDS, somatic mutations in stem cells of different tissues (muscle, intestine, and others) are in discussion for a possible role in normal or premature aging [50••].

One possible reason for genomic instability is telomere shortening which is well established in aging but also being investigated in MDS. It has been shown that it may affect all hematopoietic cells in patients with MDS. Conversely, in some MDS subtypes, like MDS with isolated monosomy 7, telomeres may be stabilized and even increase in length because of the activation of telomerase or alternative mechanisms [51]. The predominant finding of MDS and AML (75% of all cancers found in an analysis of 180 patients) in patients with Mendelian short telomere syndromes supports the fact that short telomeres are associated with aging as well as with the development of MDS [52].

One of the hallmarks of MDS development is modified epigenetic regulation affecting DNA methylation and different forms of histone modifications. While gene mutations impairing epigenetic regulation observed in MDS might not be a consequence of aging per se, these mutations arise and interact in the context of epigenetic drift—the alteration of epigenetic patterns during aging, of which the gradual decrease of global DNA methylation is the most prominent example [5, 42, 44–49, 53–58].

Frequent mutations in MDS affect the splicing machinery which in consequence impairs gene expression by splice variants and truncation of proteins during translation. Different mutations have been described, the prominent SF3B1 mutation shows a high association with a distinct morphologic (MDS with ring sideroblasts) and clinical subtype of MDS [59–61]. Several splicing defects can lead to mitochondrial dysfunction—another prominent feature of MDS biology [62]. In this context, it must be mentioned that MDS is one form of iron-loading anemia which alters energy metabolism and leads to increased ROS production [63, 64]. This may lead to further propagation of MDS and may be potentiated by age-related changes in energy metabolism.

Both mitochondrial dysfunction and deregulation of precursor mRNA splicing have been linked to other illnesses and age-related chronic diseases. Splice variants of important genes such as p53, *IGF-1*, *SIRT1*, and *ING-1* can be associated with senescence and aging. Diverse splicing defects occur in accelerated aging (progeria) and vascular aging. Changes in the activity of splicing factors and in the production of key splice variants may impact cellular senescence and the aging phenotype [23, 65].

### Altered Hematopoietic Stem Cells

These play a central part in MDS pathogenesis and aging hematopoiesis and the general role of stem cells needs to be considered. Factors that mediate selection pressure during the process of the selection of certain pools of HSCs may be similar in their effect on aging hematopoiesis and in clonal evolution—a main driver of MDS propagation [66•].

### Microenvironment and the Bone Marrow Niche

MDS affects not only the hematopoietic cells, but also the stromal niche of MDS patient aberrations can be observed. Initially, morphologic and functional changes were described and underlying molecular mechanisms are being elucidated. As in inflammation and immune mechanisms, an interplay between microenvironment and stem cells is propagating the disease by impaired stem cell supporting functions. An aging microenvironment with distorted cytokine—receptor interplay, regulation of homing, and effects on HSC age may affect both normal and clonal stem cells leading to the MDS phenotype [35•, 36]. Likewise age-related inflammatory changes in the niche can lead to ineffective hematopoiesis as described in a murine model mimicking del(5)q MDS [67–71].

MDS is also a disease of disturbed immune response either showing immune deficiency or increased inflammatory responses and autoimmunity. Recent studies have elucidated the role of key players in this aspect of MDS pathogenesis. Both innate and adaptive immune responses are shown to be affected. Immune activation may augment the propagation of MDS stem cells by selection pressure or MDS cells may trigger immune activation. Whereas initiating events remain still unclear, both probably interacting factors may foster MDS propagation [66•, 72••, 73–75]. In addition, the relevance of hyperinflammation in individuals with MDS is highlighted by frequent systemic inflammatory and autoimmune manifestations (SIAM), which are detected with a prevalence of 10–20% [76].

Normal aging of hematopoiesis is associated with changes in immune responses. In HSC aging, a shift to myeloid-biased differentiation is observed leading to an increase in innate immunity and reduction of adaptive immune responses. This phenomenon of immunosenescence which also includes a state of increased systemic inflammation contributes to other age-associated diseases and probably also to MDS [72••, 77, 78].

Many of the biologic features of MDS described above are mediated by alterations in signaling that are caused by mutations in or defective transcription and translation of genes for mediators and receptors [47, 50••, 55]. In MDS, there are more systems affected, namely regulation of hematopoiesis, proliferation, and inflammatory responses/activation compared to disturbed signaling of aging which mainly plays a role in inflammaging [37].

In MDS, especially during disease progression, disturbed regulation of transcription has a synergistic effect for the induction of enhanced proliferation [42] which is a prominent hallmark of cancer that has no counterpart in normal aging [18].

## Juvenile MDS

In the context of MDS and aging, it has to be mentioned that MDS may also occur in children and is then termed juvenile MDS. This finding is remarkable: No obvious association with aging is seen at first glance in these cases. MDS in children are rare entities, which are classified according to the WHO into three groups: MDS, juvenile myelomonocytic leukemia, and Down syndrome-associated myeloid leukemia [79, 80].

The biology of juvenile MDS is different from adult MDS and is often associated with genetic disorders and inherited bone marrow failure syndromes [81]. Accordingly, in approximately 30% of juvenile MDS patients, constitutional disorders may be detected. Interestingly, also genotypic alterations can be found that are associated with MDS predisposition such as mutations in the *TET2* gene. In later stages, the typical genetic changes associated with disease progression, which are also observed in adult MDS such as monosomal karyotypes or mutations of *RAS*, p53, or *WT1* can be found [82, 83].

## Is Myelodysplasia a Consequence of Normal Aging?

As described above, considerable progress has been made in deciphering the biology of normal aging, which includes the distinction of normal aging from pathologies associated with aging; additional progress has been made in describing MDS preceding states and elucidating initiation and progression of this disease. Despite these data, the provocative question, if MDS is simply a variant of the aging process, remains challenging.

The earliest answers supporting this hypothesis come from epidemiologic data with a clearly increasing incidence of MDS with age. As always, this observed correlation needs to be supported by establishing a causal relationship.

Some similarities between aging and MDS have undoubtedly been defined. Especially changes affecting hematopoiesis are suggestive for an involvement of aging in the development of a hematologic disorder. One example is aging hematopoiesis as a result of clonal selection of hematopoietic stem cells leading to an alteration of the HSC pool. Another is clonal hematopoiesis such as defined in CHIP which is recognized as a potential pre-MDS state with a continuous increase at an advanced age. CHIP has not only implications for MDS but also for other conditions or diseases associated with aging such as cardiovascular disease which further supports the connection with aging. Finally, many of the biologic features that drive the MDS process can also be observed in processes of aging (Table 2) or are key players in non-hematologic diseases of the elderly.

**Table 2:** Comparison of biologic features in MDS and Aging (for references, see the main text)

Biology of MDS and aging—a comparison		
Mechanism	MDS	Aging
Genomic instability	Initiates clonal hematopoiesis and is a driver of clonal evolution and progression in MDS	Genomic damage is associated with aging. Disorders with chromosomal instability such as Werner syndrome are characterized by a progeroid phenotype
Cellular senescence and telomere attrition	Telomere shortening as a factor for chromosomal instability	Telomere length determines the so-called replicative senescence Increase in the number of senescent cells
Epigenetic alterations	Main factor for dysregulated gene expression of relevant genes that leads to MDS phenotype of dysplasia and cytopenia	Profound impact in aging. Manipulation of the epigenome may improve age-related diseases and increase lifespan
RNA splicing	In many subtypes of MDS, RNA splicing defects confer altered protein expression patterns that in consequence produce the MDS phenotype	Alterations in RNA splicing are associated with senescence and aging Splicing defects occur in progeroid syndromes
Mitochondrial dysfunction	Affected in MDS with splicing mutations. ROS in tissue damage due to iron overload (MDS = iron loading anemia)	Results in altered multiple cellular functions. An increase in ROS impacts aging as suggested by the free radical theory
Stem cell exhaustion and selection	Selection pressure may be similar in their effect on aging hematopoiesis and in clonal evolution that is a main driver of MDS propagation	Functional defects of stem cells and selection from clonal pools result in cellular senescence Plays a role in inflammaging Impaired homing and mobilization and age-associated defects of HSC—microenvironment interaction
Stromal niche	An interplay between microenvironment and stem cells is propagating the disease by impaired stem cell supporting functions	Changes in cellular composition and function. Direct effects on stem cell aging Effect on impaired function and regenerative capacity of HSCs reflect a common denominator of aging
Inflammation/immune system	Both innate and adaptive immune responses are shown to be affected. Immune activation and hyperinflammation in MDS are highlighted by systemic autoimmune and autoinflammatory manifestations (SIAM)	Infllammaging describes a subclinical systemic sterile inflammation Inflammaging is associated with a variety of common age-associated diseases such as cardiovascular disease, neurodegenerative diseases, sarcopenia, and osteoporosis
Altered intercellular communication	Signaling alterations affect the regulation of hematopoiesis, proliferation, and inflammatory responses/activation	Inflammaging represents an essential feature of aging and of age-associated diseases
Regulation of transcription	Role in enhanced proliferation	DNA damage may lead to deregulation of gene expression and to increased transcriptional noise
Deregulated nutrient sensing	Not yet established in MDS pathophysiology	Nutrient-sensing pathways (mTOR, IGF-1) play an essential role in aging processes. Dietary restriction increases the healthy lifespan of many species
Loss of proteostasis	Not yet established in MDS pathophysiology	Proteostasis is impaired in aging processes and in age-related diseases such as neuro-degenerative disorders

On the other hand, there are clear data demonstrating that MDS is not inevitable with aging: for example, the risk of developing hematologic malignancies, particularly MDS, is higher in patients with clonal hematopoiesis than in persons without, but by far not all of them develop MDS. In addition, as far as we know, not every person contracts MDS, if he or she gets just old enough. Unfortunately, this cannot be definitively proven due to inherent methodological limitations. However, data on the incidence of MDS and leukemia and observation of elderly persons not contracting MDS support the notion that age alone does not inevitably lead to MDS, i.e., without any further initiating, probably age-independent factors.

In addition, juvenile MDS is an example that MDS might develop in early life years, although it must be stated that the biology of juvenile MDS is quite different from MDS in the elderly. This is not so clear in secondary MDS that may be induced by radio- or chemotherapy also in younger persons and shares biologic similarities with primary MDS [84].

**Fig. 1:**
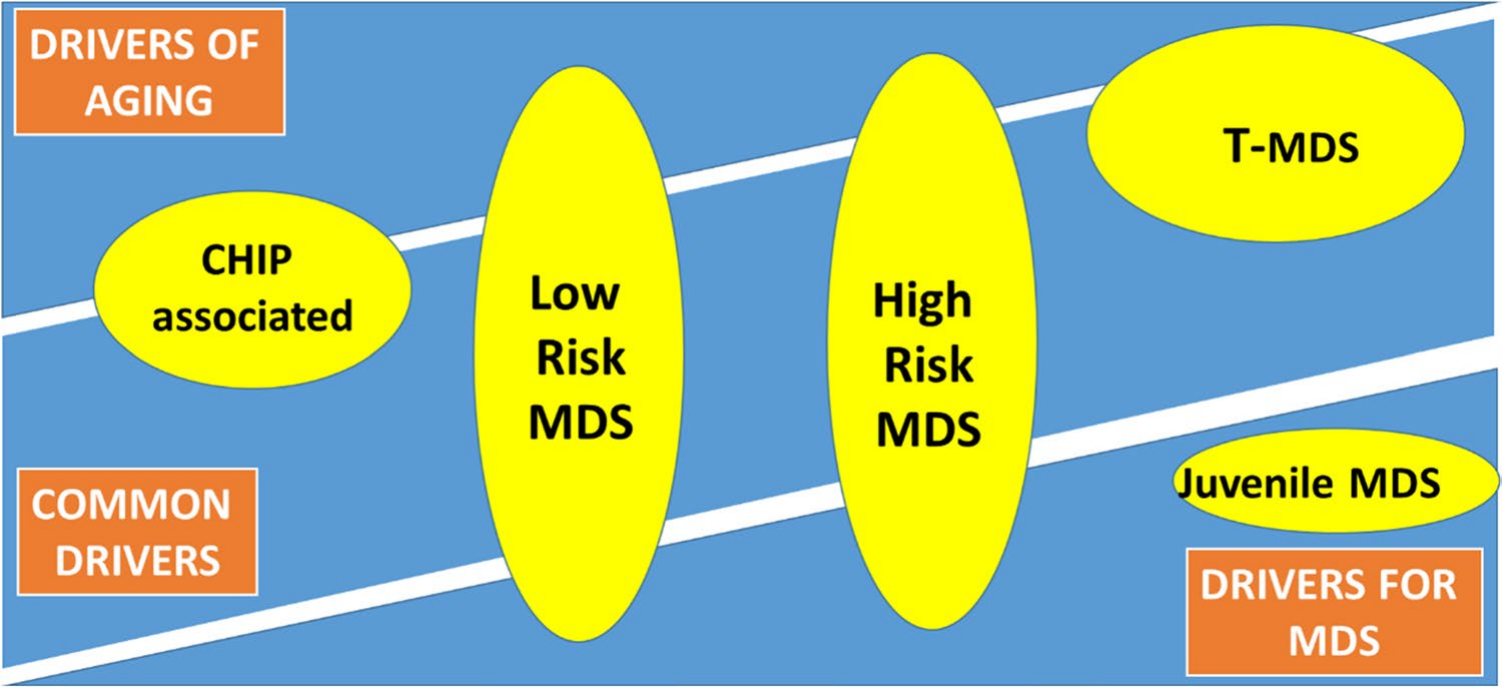
MDS as the interplay of a clonal disease and of normal and premature aging. Age-related factors, MDS-related factors, or both are contributors to the development of MDS. Aging may act as the main driver or promotor of MDS that may also arise independently from age. Different subtypes or disease entities of MDS are distinctively affected by aging

A possible solution for this conundrum is the notion that aging certainly contributes to the development of MDS. One might hypothesize that in many cases aging is the main driver of MDS, whereas in others aging promotes the specific phenotype. MDS might thus be seen as an interplay of clonal disease and normal or premature aging. Probably different subtypes or disease entities of MDS are distinctively affected by aging. Apart from secondary or juvenile MDS mentioned above, this might be the case with MDS as separated by risk categories. Lower risk MDS might have a higher association with aging both in genotype (more CHIP-associated mutations) and in clinical manifestations (e.g., fatigue), whereas higher risk MDS is probably more driven by less age-dependent factors including p53 mutations or monosomy 7 [85–87]. An attempt to visualize this hypothesis is shown in Fig. 1.

Also, for other diseases besides MDS, this interplay between hallmarks of aging and disease-specific factors may play an important role in disease initiation, propagation, and timing during a lifetime. The correlation with the hallmarks of cancer is described above; in addition, common mechanisms for malignant and nonmalignant age-associated diseases were mentioned. In hematology besides MDS, age-dependent alterations may prepare the ground for the development of B chronic lymphocytic leukemia (B-CLL), which also shows an increasing incidence with age. Performing a comprehensive proteome analysis, primary human B-CLL cells and B-cells from younger and elderly healthy donors showed a distinct proteome signature suggesting that these age-associated proteome alterations may represent a signature of inflammaging and immunosenescence and may mediate an increased risk for the development of B-CLL [88].

## Conclusion and Future Directions

The relationship of MDS with aging is meanwhile well established. Without any doubt, biological processes also active in aging are key players in initiation and propagation of the disease. For clinicians, the consideration of aging in the management of MDS patients is of high importance. Future and already ongoing research addresses the question if age-related changes that initiate or propagate the development of MDS are amenable to intervention. In addition, concomitant and driving changes due to aging should be taken into account when developing new treatment approaches for MDS.
